# Biosimilar medicines: creating sustainable competition in an era of a new patent cliff in biological medicines

**DOI:** 10.1186/2052-3211-8-S1-P28

**Published:** 2015-10-05

**Authors:** Maarten Van Baelen

**Affiliations:** 1 European Generic and Biosimilar Medicines Association, Brussels, 1000, Belgium

## 

For many years European governments have sought to ensure a high degree of competition in off-patent pharmaceutical markets in order to generate price competition - and consequently benefits such as improved patient access or savings for payers after patent expiry. The pharmaceutical industry believes that access to valuable new treatments and post-exclusivity competition are essential for the sustainability of healthcare systems.

Biological medicines have become increasingly important over the last years. Twenty-seven per cent of pharmaceutical sales in Europe come from biological medicines. This market grew by 5.5% vs. a total market growth of 1.9% in value sales between 2012 and 2013. Many of Europe's top selling biologic molecules are facing patent expiry by 2020[1].

Most biological medicines come at a high cost and governments have difficulty in coping with these costs in their constrained pharmaceutical budgets, especially in current times of austerity. To date, biosimilars account for less than 0.5% of the $221 billion market of biological medicines worldwide. Biosimilars can bring huge savings for payers, and will increase the access to medicines for patients who could not otherwise afford treatment[2].

**Figure 1:**
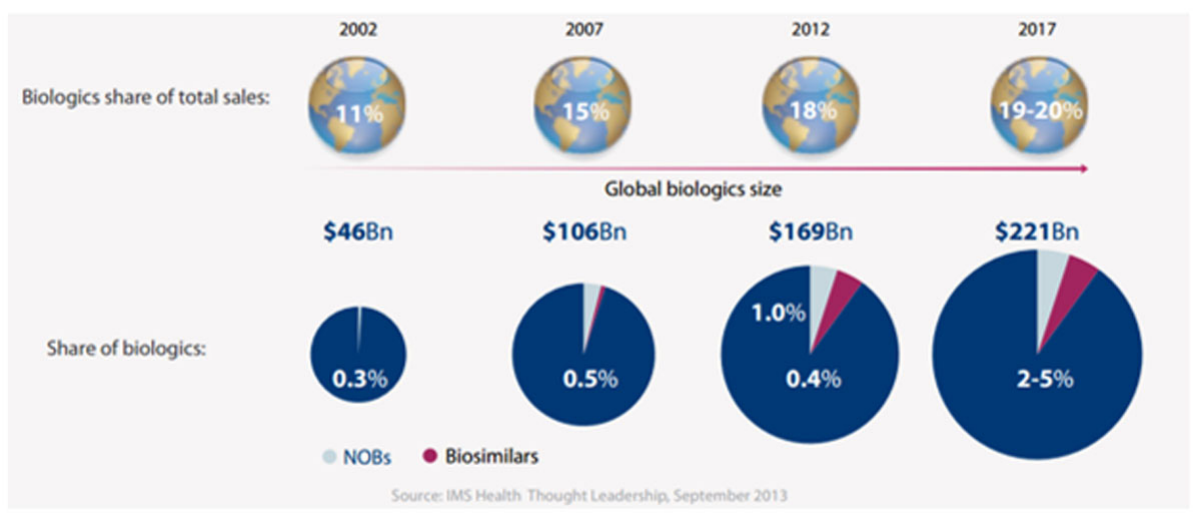
The biologics Market - IMS Health Thought Leadership, September 2013

Governments must realize that biosimilar medicines are different to generic medicines and as such a unique approach is needed. By applying the generic pricing model to biosimilar medicines, governments risk marking the biosimilar market unsustainable and patients and payers will no longer benefit.
